# The turn of the dice: Patrick Hughes’ Hollow Dice and Reverspectives

**DOI:** 10.1177/20416695231165623

**Published:** 2023-04-28

**Authors:** Brian Rogers, Patrick Hughes

**Affiliations:** Department of Experimental Psychology, 6396 University of Oxford, Oxford, UK; Reverspective, London, UK

**Keywords:** depth, 3D perception, binocular vision, motion, perception, stereopsis

## Abstract

Patrick Hughes’ *Reverspectives* demonstrate the importance of perspective as a source of information about the structure and layout of the three-dimensional (3D) world. More recently, he has created a new work of art—*Hollow Dice—*in which the actual concave structure of the dice is seen as *convex*. In this article, we examine the similarities and differences between these two perceptual phenomena as well as attempting to explain how and why they arise. Popular interest in both effects is based on the fact that “*what we perceive*” does not correspond to “*what the reality is*.” As a consequence, *Reverspectives* and *Hollow Dice* are often categorized and labeled as “illusions.” However, if we consider the *information* that is available in patterns of light reaching our eyes—rather than the “actual” 3D structure of the *Reverspectives* and the *Hollow Dice—*we are in a better position to explain how the size, the viewing distance, the perspective features, the convexity bias, and observer movements determine what we see when viewing these novel and fascinating visual effects.

## Background

**Figure 1.**
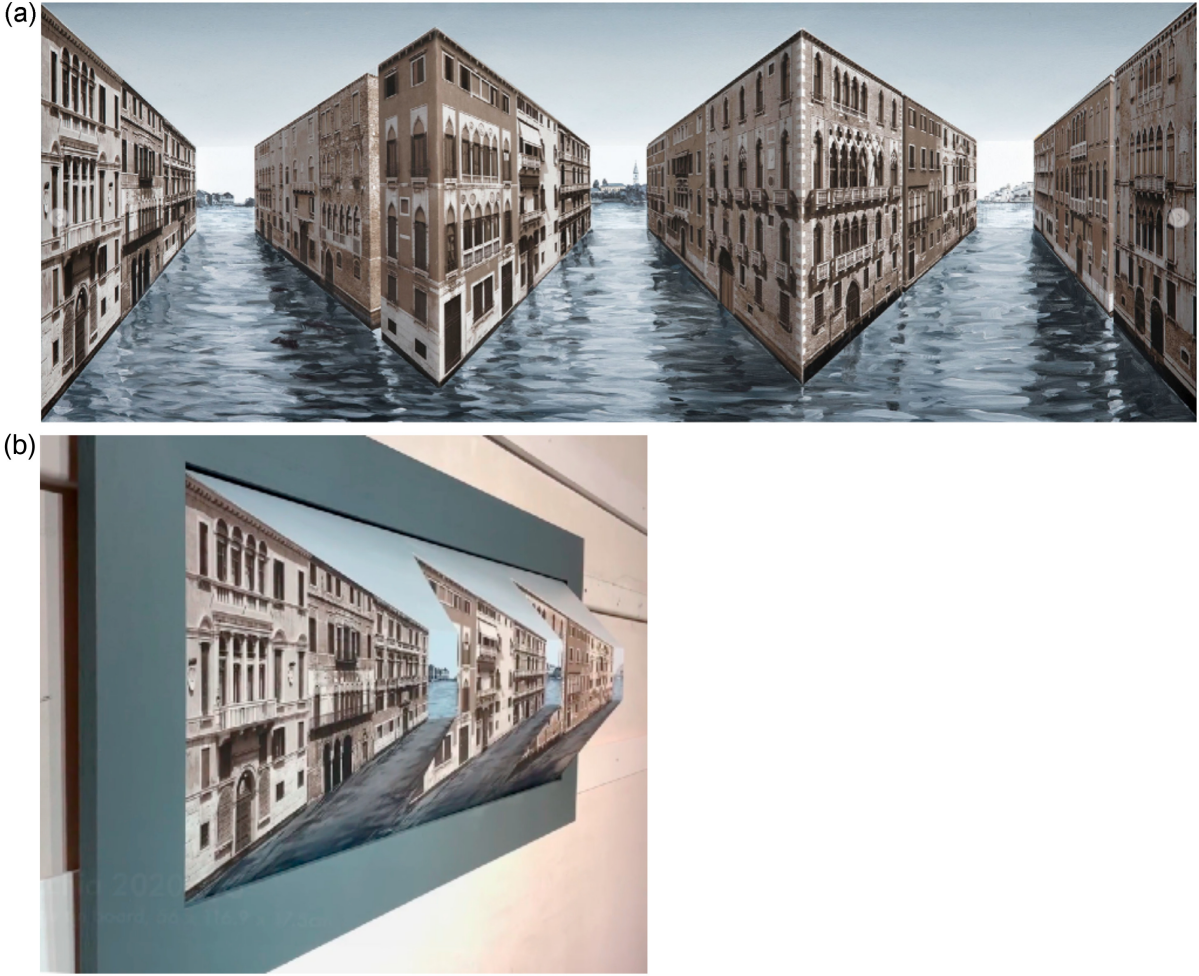
(a) A frontal view of Patrick Hughes’ 2019 Reverspective “*Citta Vecchia.*” The physical structure consists of three *protruding* truncated pyramids. (b) The same Reverspective seen from a moving camera to reveal the actual 3D structure.

One of us (PH) is best known for his three-dimensional (3D) Reverspective art, in which the 3D scenes appear to recede into the distance, whereas in reality, the actual 3D structure stands out in front of the background (Figure 1a and b) (Hughes, 2018). Moreover, when the observer moves from side-to-side (or bobs up-and-down), the 3D scene appears to rotate so as to follow the observer's movements—something that normally never happens when we view the 3D world. First and foremost, Reverspectives are works of art with intriguing and imaginative content. However, from a vision scientist's point of view, Reverspectives have proved to be a very useful tool for investigating human visual perception. In particular, Reverspectives have revealed the role and importance of both perspective and motion parallax in 3D vision. For example, Papathomas (2002), Papathomas and Bono (2004), and Rogers and Gyani (2010) have shown that the perspective information in a Reverspective overrides the binocular disparities unless the observer is very close (between 50 and 100 cm) to the artwork. In addition, the same authors have shown that the perception of reversed depth can be observed in a variety of different 3D scenes from a simple grid of converging lines to natural scenes (e.g., Rogers & Gyani, 2010, Figure 5).

## Hollow Dice

**Figure 2.**
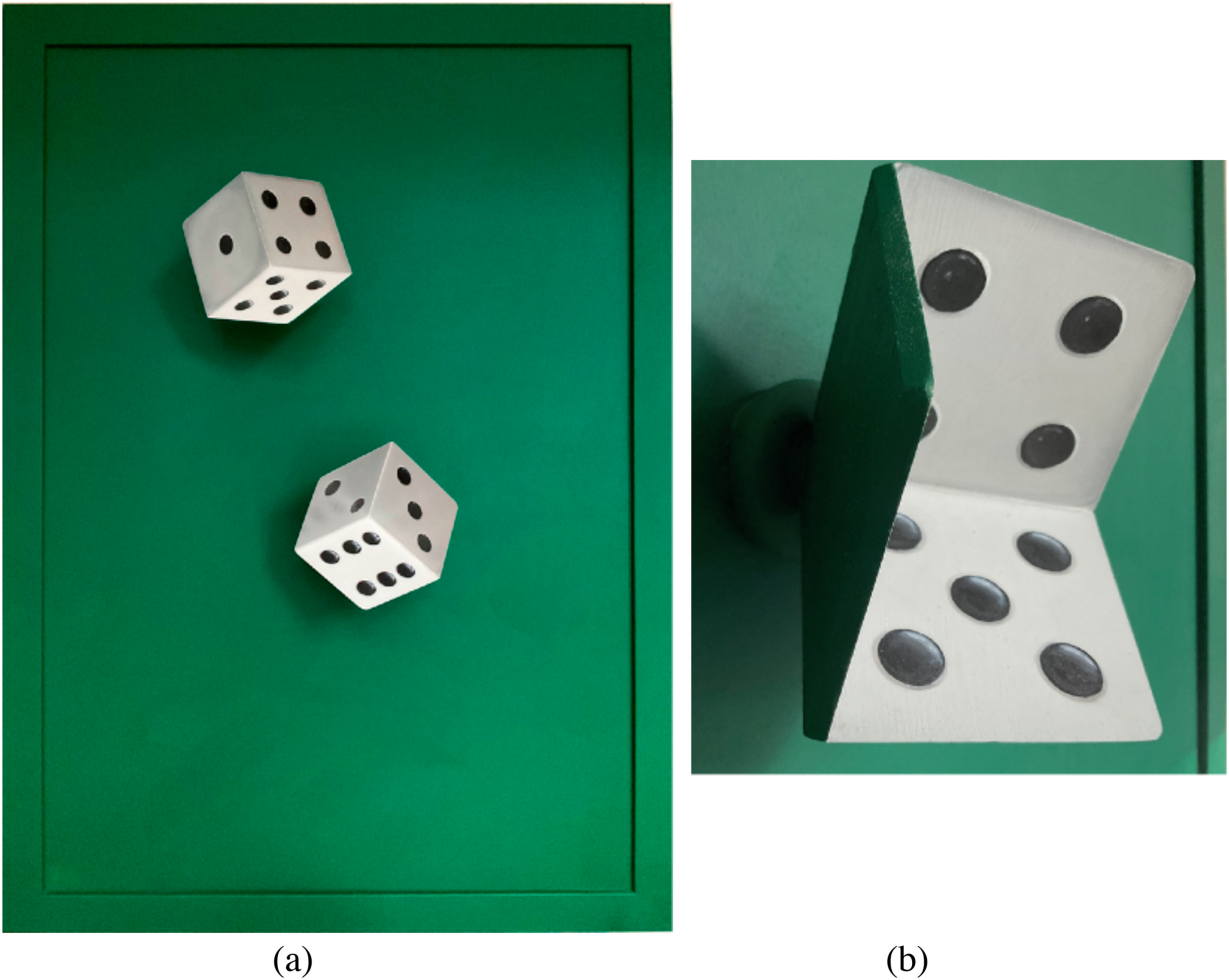
(a) Patrick Hughes’" Hollow Dice.” (b) The same “Hollow Dice seen from a moving camera to reveal the actual concave structure of the dice.

**Figure 3.**
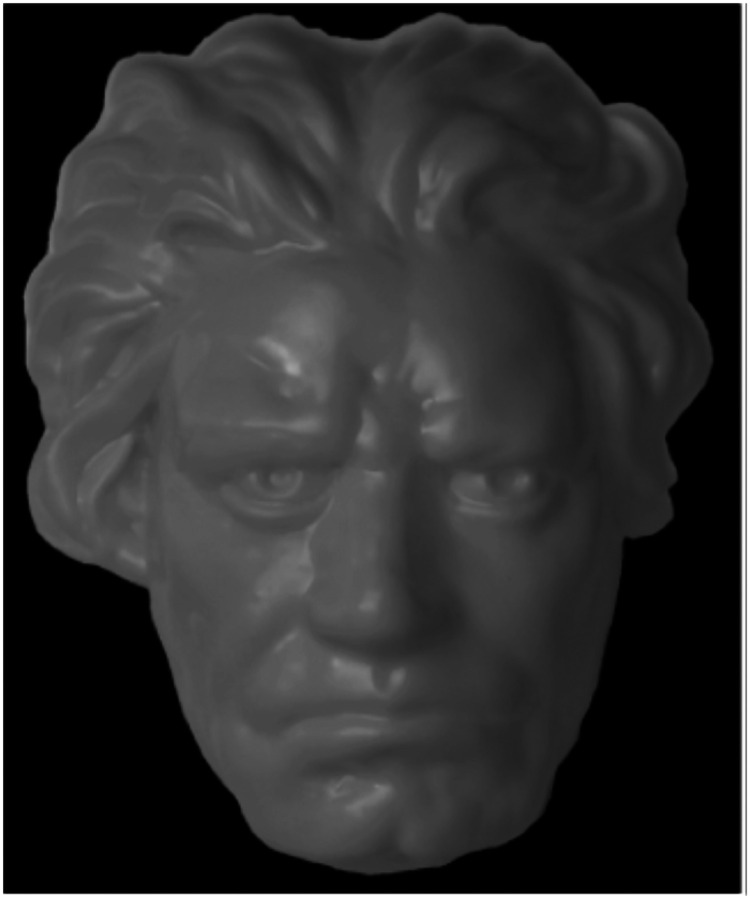
A photograph of a hollow face (from Rogers, 2017a).

In 2022, one of us (PH) created a new work of art that consists of a pair of hollow dice that stand out in front of a plain background (Figure 2a). The Hollow Dice have been exhibited at the Messmer Gallery in Riegel, Germany, the Adelson Gallery in New York, and the ECVP2022 meeting in Nijmegen. Although the Hollow Dice have a concave (hollow) structure (Figure 2b), they are seen as convex cuboids (like regular dice), unless the observer is very close. Moreover, when the observer moves from side-to-side (or bobs up-and-down), the dice appear to turn or rotate so as to follow the observer's movements. (Hence the title of this paper: “*The Turn of the Dice*”). These perceptual consequences reveal an interesting similarity between Hughes’ Reverspectives and his Hollow Dice but there is an important difference. In Reverspectives, the actual 3D structure is largely *convex* (i.e., the pyramids protrude out towards the observer, Figure 1b) but the scene appears to recede into the distance, whereas for the Hollow Dice*,* the actual 3D structure is *concave* but the dice appear to be convex. These differences suggest that the Hollow Dice might have more in common with the hollow face effect (Figure 3) that has been described and extensively investigated over many years (e.g., Brewster, 1826; Gregory, 1970; Wade et al., 2003; Wallace Wallin, 1905).

## Hollow Faces

Richard Gregory's explanation of the hollow face effect is based on the realization that all of us have had a lifetime of exposure to normal, convex faces and hence the perception of a hollow face is a very unlikely “perceptual hypothesis” (Gregory, 1980). Evidence in favor of Gregory's explanation comes from the finding that observers are more likely to see an *upright* hollow face as convex (i.e., reversed in depth) than they are an *upside-down* hollow face (Kohler, 1940; Papathomas, 2017). Hill and Bruce (1993) have suggested an alternative explanation based on the statistics of the visual world in which we have evolved—that is, there are more convex objects than concave structures. Evidence in favor of Hill and Bruce's explanation comes from the finding that it is not just hollow faces that are seen as convex but rather that the majority of the hollow moulds of both familiar and unfamiliar objects are also seen as convex (Hill & Bruce, 1994). In other words, there does not seem to be anything special about faces. As a result, it could be argued that the visual system's bias towards convexity (based on the statistics of the world) might explain why the Hollow Dice are seen as convex. But there are important differences between Hollow Dice and hollow faces. The information about the 3D structure of faces is based almost entirely on the patterns of shading, as can be seen in Figure 3. In addition, (i) facial images contain relatively few steep luminance gradients (contours) that are favored by the disparity system (Bülthoff & Mallot, 1988) and (ii) there is an absence of both texture gradients and linear contours in facial images and therefore little or no perspective information.

## Hollow Dice

Hughes’ Hollow Dice differ from hollow faces in both these respects. The contours between the sides of the hollow dice provide binocular disparities (which should support a concave “interpretation”^1^) as well as some (limited) perspective information. In his book “The Intelligent Eye,” Richard Gregory (1970) showed that an *actual* wire-frame Necker cube looks cuboid in shape even though the far side of the cube subtends a smaller visual angle. In other words, there must be size scaling, driven by the difference in the distance between the near and far sides of the cube, in order to compensate for the difference in angular subtense of the near and far sides of the cube. In addition, the contours linking the near side to the back side of the cube *converge* in the retinal projection. However, if an observer manages to reverse the depth order so that the far side of the cube is perceived as being closer, the wire-frame Necker cube looks like a truncated pyramid because the size-scaling is based on the incorrectly perceived distance (Gregory, 1970).

It follows that if the Hollow Dice have a regular cuboid shape, those dice should suffer from a similar perceptual distortion whenever the hollow dice are seen as reversed in depth. And this is what we found—the dice appear to have a distorted cuboid shape. On the other hand, if the Hollow Dice are given a small amount (10%) of perspective distortion so that contours between the three sides actually *diverge* from the front to the back of the cube (Figure 2b), the distortion is not seen. In this case, the depth-reversed Hollow Dice appears to have a regular cuboid shape, as can be seen in Figure 2a. These observations provide good evidence that perspective information affects the perception of 3D *shape* in the Hollow Dice—that is, the absence or presence of the perspective distortion influences whether the dice are seen as regular or distorted cuboids. And note that the perspective information provided by the diverging contours of the dice is further enhanced by the (limited) texture gradient on the sides of the dice. The side view of the hollow dice (Figure 2b) shows that the spots on the sides of the dice *increase* in both their sizes and their spacing toward the back of the dice, which is consistent with the perspective of the *diverging* contours.

## Depth Reversals

These observations raise two interesting empirical questions: first, while it is true that the presence of *cuboid* or *distorted* perspective in the Hollow Dice affects the perceived 3D *shape* of the dice, does the choice of perspective affect whether the dice are *seen* as convex or concave? Second, given that the contours and the spots on Hughes’ dice provide unequivocal disparity information specifying the hollow shape of the dice, does this make it *more likely* that the dice are seen for what they are (i.e., concave^2^), when compared with the perception of a hollow face? To answer the first question, we tried approaching the dice with either a *cuboid* perspective or a 10% *distorted* perspective and recorded the “flipping^3^” distance when the appearance changed from convex to concave (hollow). The results: there was little or no difference in the “flipping” distance (∼50 cm) when the dice were approached using one eye. With binocular viewing, there was also little or no difference in the distance (between 120 and 150 cm) at which the percept changed from convex (reversed depth) to concave (veridical depth) as a function of whether the dice had *cuboid* or *distorted* perspective. However, the “flipping” distance at which the percept flipped from convex to concave during the approach was *greater* (i.e., farther from the dice) with binocular viewing (in both cases), as might be expected given the presence of disparities that signal the actual, concave shapes the dice.

The second question—comparing hollow dice to hollow faces—is a more difficult question to answer since there are many different parameters of both stimuli—for example, their size, texture, color, and so on. It seems very likely that the perceived reversal in depth, which is a common feature of both hollow dice and hollow faces, must be due to the visual system's “preference” or statistical bias for seeing convex over concave forms. So what are the predictions? Under binocular viewing, the *absence* of significant disparate contours on the hollow faces, and the *presence* of disparate contours on the hollow dice, should mean that the “flipping” distance should be *smaller* (i.e., closer) when approaching a hollow face than when approaching the hollow dice. Under monocular viewing, the *absence* of perspective in the hollow face, and the *presence* of limited perspective in the hollow dice might suggest that the “flipping” distance would also be *smaller* (i.e., closer) when approaching a hollow face, than when approaching the hollow dice. In other words, the perceptual reversal in both cases would be stronger and more resistant to change. Consistent with those predictions, we found that the “flipping” distance was typically *smaller* (i.e., closer) for most observers in the approach to a hollow face than to the Hollow Dice under both monocular and binocular viewing conditions.^4^

## The Role of Observer Movement in Reverspectives

**Figure 4.**
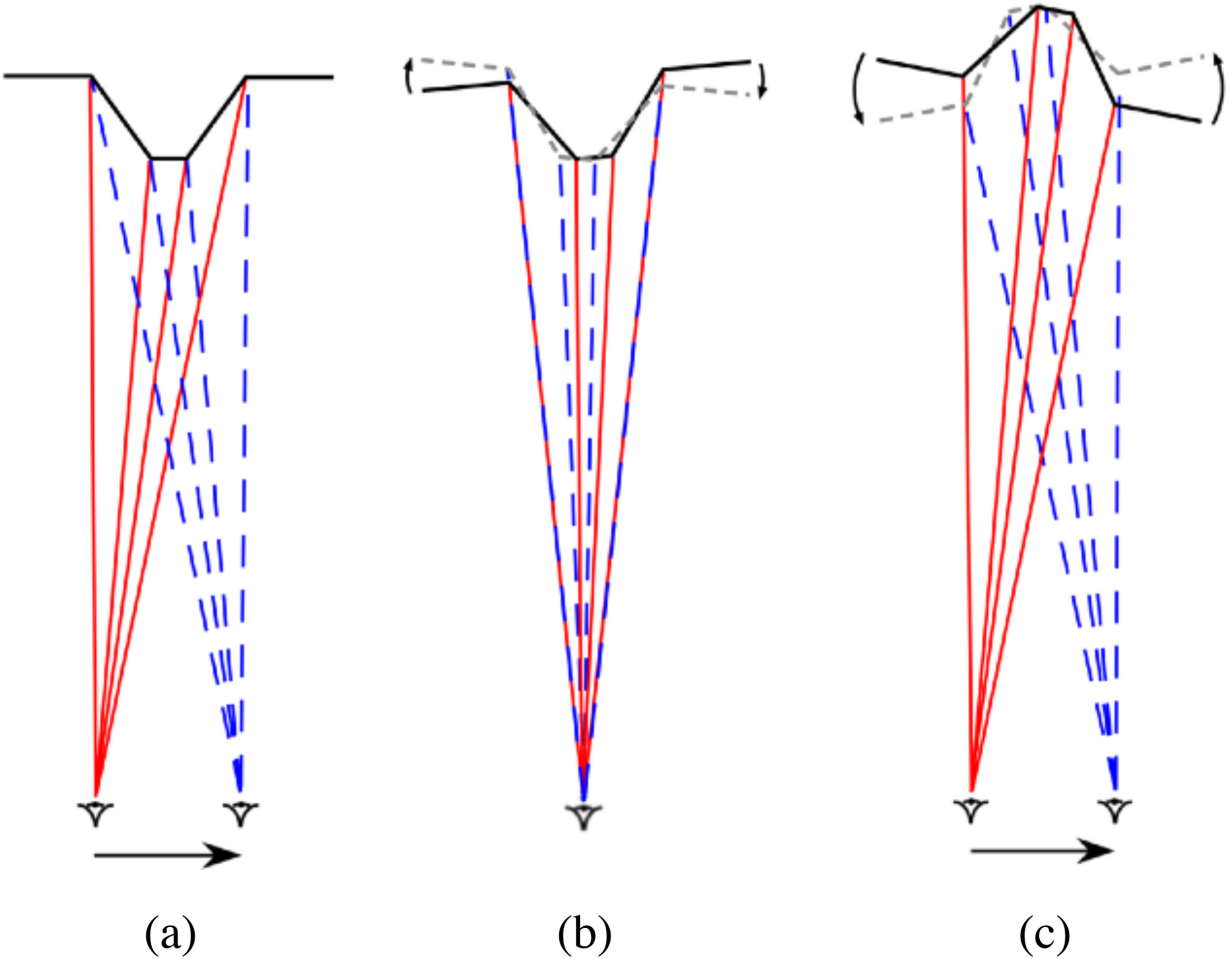
(a) A plan view of the eye of a *monocular* observer moving from left-to-right while viewing a *protruding* truncated pyramid (as in a Reverspective). (b) The parallax transformation created by that observer’s movement is equivalent to a *clockwise* rotation of the pyramid with respect to the observer's *line-of-sight*. (c) The same parallax transformation is also created when the observer moves left-to-right while viewing a perceptual, depth-inverted truncated pyramid (as a result of the perspective information on the sides of the pyramid), such that it appears to rotate in the same direction, that is, *counter-clockwise* with respect to the observer's line-of-sight. The predicted angle of counter-clockwise rotation is exactly twice the angle of the observer's rotation around the three-dimensional (3D) structure (based on a small angle approximation) (figure adapted from Rogers, 2016). [Note that the two superimposed optic arrays (red and blue) depicted in (b) are the *same* as the two optic arrays from different vantage points depicted in (a).]

There are two additional questions about the depth reversals seen in all three situations. First, why should Reverspectives, Hollow Dice, and the hollow faces all appear to rotate *with* the movements of the observer's head when the perceived depth order is reversed? Second, why should the perceived depth be *enhanced* when the observer makes side-to-side (or up-and-down) head movements? For any 3D structure or scene, side-to-side movements of the head produce motion parallax—the relative motion in the optic array (or retinal image) between parts of the object or scene at different distances from the observer (Rogers & Graham, 1979; Von Helmholtz, 1910). Assuming fixation on a particular point in the scene, the images of parts of the object or scene that are closer to the observer will move in the *opposite* direction to the observer's head movements and the images of parts of the object or scene that are more distant from the observer will move in the *same* direction as the observer's head movements. For example, if a monocular observer moves from left-to-right while viewing a protruding wedge or truncated pyramid (like those in a Reverspective, Figure 4a), the perceived object or scene effectively rotates^5^ in the opposite direction—that is, *clockwise—*with respect to the observer's *line-of-sight*^6^ (Figure 4b).

However, if the perceived depth is *reversed*, the parallax transformation remains the same but the parts of the object or scene that are seen to be farther from the observer will move in the *same* direction as the observer's head movements, and parts of the object or scene that are closer to the observer will move in the *opposite* direction to the observer's head movements. The only situation that is compatible with this state of affairs is to see the 3D object rotate (counter-clockwise) *with* the observer's movements (Figure 4c). And this is what observers report. This might sound unlikely since objects do not typically move or rotate when we move our heads but this is what we perceive when viewing a Reverspective from a distance.

## The Role of Observer Movement in Hollow Faces and Hollow Dice

**Figure 5.**
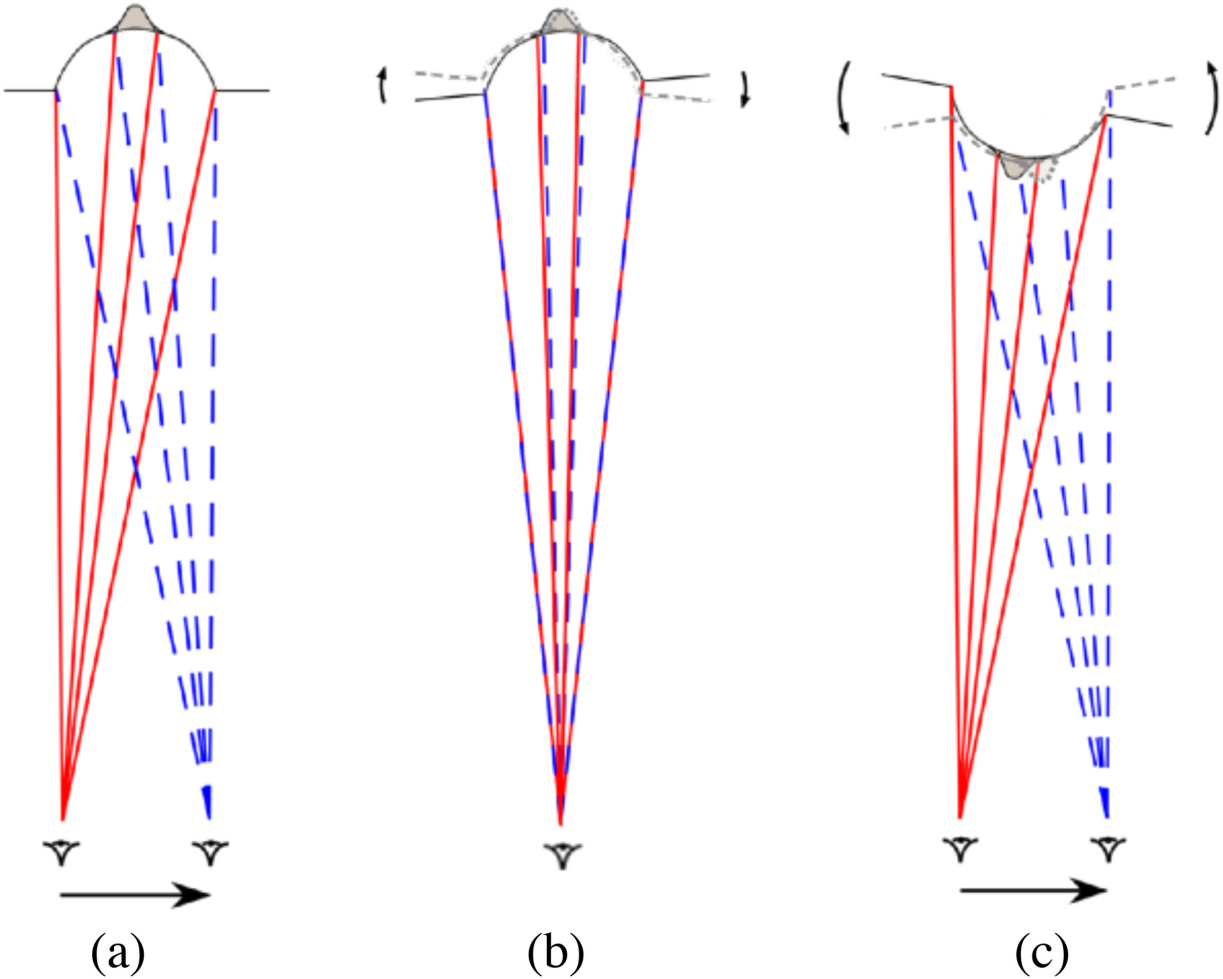
(a) A plan view of the eye of a *monocular* observer moving from left-to-right while viewing a *hollow* face. (b) The parallax transformation created by the observer’s movement is equivalent to a *clockwise* rotation of the face with respect to the observer's line-of-sight. (c) The same parallax transformation is also created when the observer moves left-to-right while viewing a depth-inverted hollow face (i.e., convex) that rotates in the same direction: *counter-clockwise* with respect to the observer's line-of-sight (the predicted angle of *counter-clockwise* rotation is exactly twice the angle of the observer's rotation around the three-dimensional (3D) structure based on a small angle approximation). [Note that the two superimposed optic arrays (red and blue) depicted in (b) are the *same* as the two optic arrays from different vantage points depicted in (a)]

In Reverspectives, the physical structure of the truncated pyramids and wedges protrudes toward the observer (Figure 1b), that is, the structure is actually *convex* but the scene is seen to recede into the distance (i.e., *concave* in each of the pyramids). What about the hollow faces and Hughes’ Hollow Dice in which the 3D structure is actually *concave* but is perceived to be *convex*? The analysis is essentially the same. When the observer moves from left-to-right while viewing a hollow face (as shown in Figure 5a), the parallax transformation created by the hollow face is equivalent to a rotation of the hollow face in a *clockwise* direction with respect to the observer's *line-of-sight* (Figure 5b). However, if the perceived depth is reversed and the face is seen as *convex*, the only situation that is compatible with the parallax transformation is to see the convex face rotate in a counter-clockwise direction, that is, *with* the observer's movements (Figure 5c). And this is what observers report.

What do observers see when viewing the Hollow Dice? At the ECVP2022 meeting in Nijmegen, several dozen volunteers were asked to approach the Hollow Dice from a distance of 3 to 4 m. Viewing was *either* monocular *or* binocular. Before approaching the hollow dice, all observers reported that the dice appeared to be depth-reversed, that is, *convex* like normal dice. As they approached the Hollow Dice with one eye closed, the majority of observers continued to see the dice as *convex* up to the point when they were very close—typically < 50 cm from the dice.

Using two eyes, (which provide disparity information to specify the actual concave 3D structure of the dice), observers typically reported that they continued to see the dice as *convex* up to the point that they were about 100–150 cm away. In other words, the pattern of results, including the appearance of the depth and the subsequent reversal of depth as observers approached the Hollow Dice, is similar to that seen with hollow faces.

## The Role of Motion Parallax in Reverspectives, Hollow Dice, and Hollow Faces

**Figure 6.**
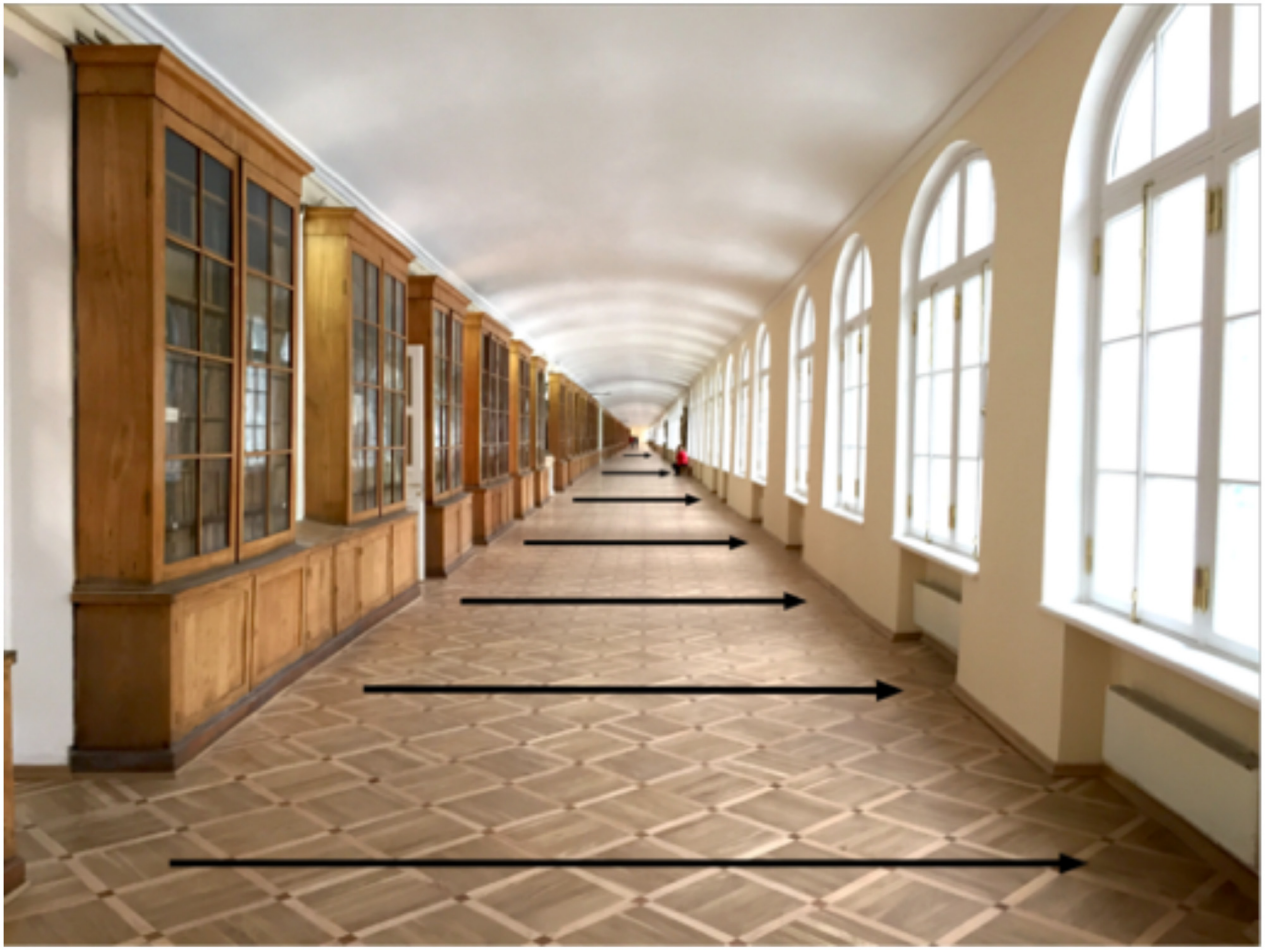
When the observer moves from right-to-left (or vice versa) while fixating on the far end of the corridor, there is a gradient of motion (motion parallax) from near-to-far. The texture elements of the floor of the corridor create a gradient of texture from near-to-far that exactly maps onto the gradient of motion (adapted from Rogers, 2017a).

What effect does the motion parallax created by the observer's side-to-side movements have on the perceived depth in each of the three situations—Reverspectives, Hollow Dice, and hollow faces? In all three cases, observers typically report that the amount of perceived depth is *greater* and more *vivid* when the observer makes those side-to-side or up-and-down head movements (Rogers, 2017b). Why might this be the case? The perspective gradients that are present on the flanks of the truncated pyramids of a Reverspective (Figure 1b) are a consequence of *projective geometry—*in both the texture gradients and converging contours of the depicted scene. But note that the motion parallax transformations created by observer movement are also a consequence of *projective geometry*, as can be seen in Figure 6.

When the observer moves from the right-to-left, the gradient of parallax motion (as indicated by the length of the arrows) exactly maps onto the gradient of the size of the texture elements on the floor. The two sources of information necessarily *complement* each other in both direction and magnitude and this is true for all the surfaces in the scene. Hence, it is not clear that the size and motion gradients should be treated as if they were separate and independent sources of information. The results from the viewing of Reverspectives suggest that the perspective gradients on the sides of the truncated pyramids and wedges determine the *sign* of the depth structure (i.e., from near-to-far) and the sign of the depth then determines how the parallax information is “interpreted” or utilized.

The hollow face effect is quite different from Reverspectives. Typically, there is no perspective information and, as pointed out previously, the perceived depth of the face is a consequence of (i) the shading information and (ii) the bias in seeing the depth structure as convex rather than concave. But the perceptual consequences are similar. The perceived depth of a depth-reversed hollow face sets the *sign* of the depth structure (i.e., convex) and this then determines how the parallax information is “interpreted” or utilized. The Hollow Dice shares features that are common to both Reverspectives and hollow faces. As mentioned earlier, the converging contours between the three sides of the dice and the sizes and the spatial separation of the spots on the sides of the dice provide some (limited) perspective information about the 3D *shape* of the dice and this affects the shape the observers see. However, the perspective information in Hollow Dice is minimal^7^ compared with the extensive perspective of the scene in a Reverspective. As a consequence, it is unlikely that the perspective information plays a significant role in determining the *sign* of the depth structure of the dice. Instead, it is the bias for seeing the depth structure of the Hollow Dice as *convex* that sets the *sign* of the depth structure and this then determines how the parallax information is “interpreted” or utilized.

## Motion Parallax as a Special Case of the Kinetic Depth Effect (KDE)

The geometric analyses shown in Figures 4 and 5 show how it is possible for observers to see the 3D structure of both Reverspectives and Hollow Dice as rotating *with* the side-to-side or up-and-down movements of the observer, even though this is something that rarely (if ever) occurs in the real-world. Why should this be possible? As can be seen in Figures 4b and 5b, the parallax transformation created when an observer moves from left-to-right is equivalent to a *clockwise* rotation of the object or scene with respect to the observer's *line-of-sight*. In this case (seeing the Reverspective correctly as a protruding structure, or the hollow face correctly as concave), the parallax is consistent with the object or scene remaining *stationary* with respect to the *world*. The fact that observers see the object or scene rotating in the *same* direction as the side-to-side movement of the observer when the depth is reversed—that is, seeing a Reverspective as receding and a hollow face as *convex* (Figures 4c and 5c),—suggests that there is no “stationarity” assumption or constraint in the visual system (Rogers, 2016). In other words, the visual system is “content” to see a scene or a face rotating during observer movement.

Why should this be the case? In 2016, Rogers suggested that motion parallax is better understood as a special case of the KDE (Wallach & O'Connell, 1953), in which the perceived depth structure is ambiguous but the depth order and the direction of rotation are always linked together (Rogers, 2016). For example, a rotating semi-transparent sphere covered with dots can be seen as rotating in one direction with one particular depth order or rotating in the opposite direction with the opposite depth order. If motion parallax is treated as a special case of a KDE (with rotation through a small angle—Figure 4b), the *depth order* information (which is provided by the perspective in Reverspectives, and the shading and convexity bias in hollow faces and Hollow Dice) is necessarily *coupled* with a particular direction of rotation*.* As a consequence, once the depth *order* information is established, the object or scene is constrained to rotate in a particular direction with the observer's movements. And this is what observers see.

Why should the visual system treat the parallax transformations as if they are a variant of the KDE with a small angle of rotation? Or, to ask the question a different way, why is it not possible for the visual system to use the *proprioceptive signals* that accompany a head movement to determine how the parallax signals should be “interpreted”? In principle, those proprioceptive signals should be capable of uniquely specifying the observer's movements and hence the direction of the observer's rotation around the object in the scene—for example, if the observer moves to the right, the object should appear to rotate *clockwise* with respect to the *line of sight* (Figures 4b and 5b). One answer is that motion parallax is also produced when an object translates with respect to the observer. Rogers and Graham (1979) referred to this as *object-produced* parallax, as opposed to *observer-produced* parallax. If a 3D object translates in a straight line across the observer's line of sight, the parallax transformation produced by the 3D object once again signals the rotation of the object with respect to the observer's line-of-sight. For example, the parallax transformation of an object translating along a straight line trajectory from right-to-left creates a *clockwise rotation* of the object with respect to the line of sight. But objects do not necessarily translate along straight-line paths. For example, a 3D object moving along a curved path that is at a fixed distance from the observer's eye will not create any motion parallax. Hence, an *object-produced* parallax transformation only provides information about how the object rotates with respect to the *line-of-sight*, rather than the object's orientation with respect to the world. And it is this rotation with respect to the line-of-sight that we see when viewing Reverspectives, Hollow Dice, and in hollow faces.

## Does Size Matter?

The word “perspective” is used in a variety of different ways that include the photographic and artistic techniques that can be used to give the impression of depth and distance. But the word “perspective” also has a formal definition based on the geometry first described by Euclid in his book “*Optics.*” An approximate translation of Euclid's 5th postulate states that:

The (angular) size of the image of an object is inversely proportional to the distance of the object from the eye (Howard & Rogers, 1995, p. 5).

An approximate translation of Euclid's 6th postulate states that:

The angular velocity of an object moving at constant linear velocity is inversely proportional to its distance from the eye (Howard & Rogers, 1995, p. 5).

These facts of projective geometry are best appreciated by considering the *optic array* at a given vantage point, that is, a description, in *angular* terms, of the patterns of light reflected off the multitude of surfaces that surround us in the world (Gibson, 1979). As a consequence, the angular separation between lines that are parallel in the world is not constant in the optic array; surfaces in the world create gradients of texture; and motion parallax is created when the vantage point moves. For a visual scientist, it is an empirical question as to whether the human (or any other) visual system uses any or all of these geometric consequences.

On the other hand, artists over the centuries have successfully exploited the various consequences of geometric perspective in their flat paintings and drawings. The success of those flat paintings and drawings in giving the impression of depth and distance provides good evidence that the human visual system is able to use that information. Reverspectives are unique in the art world in that the perspective information is presented on the sides of the truncated pyramids and wedges that extend out of the flat canvas. Take Hughes’ “*Citta Vecchia*” Reverspective as an example (Figure 1). The angular subtense of the near end of the row (at the bottom of the pyramid) is approximately^8^ four times the angular subtense of the far end of the row of buildings (at the apex of the pyramid). This provides potential information that the far end is four times as far away than the closest end.^9^ And Reverspectives reveal that this perspective information is sufficient to override the disparity information that signals the opposite depth relationship.

But what happens when an observer approaches a Reverspective? The angular size of both the far and near ends of the row of buildings will increase but because the far end is physically closer (at the top of the pyramid), it will increase at a faster rate. In other words, as an observer approaches a Reverspective, the perspective information lessens but, at the same time, the disparities that specify the “real” (protruding) shape of the truncated pyramids and wedges *double* with every halving of the viewing distance. As a consequence, the increased disparities eventually override the perspective information and the observer will see the true 3D structure of the artwork.

**Figure 7.**
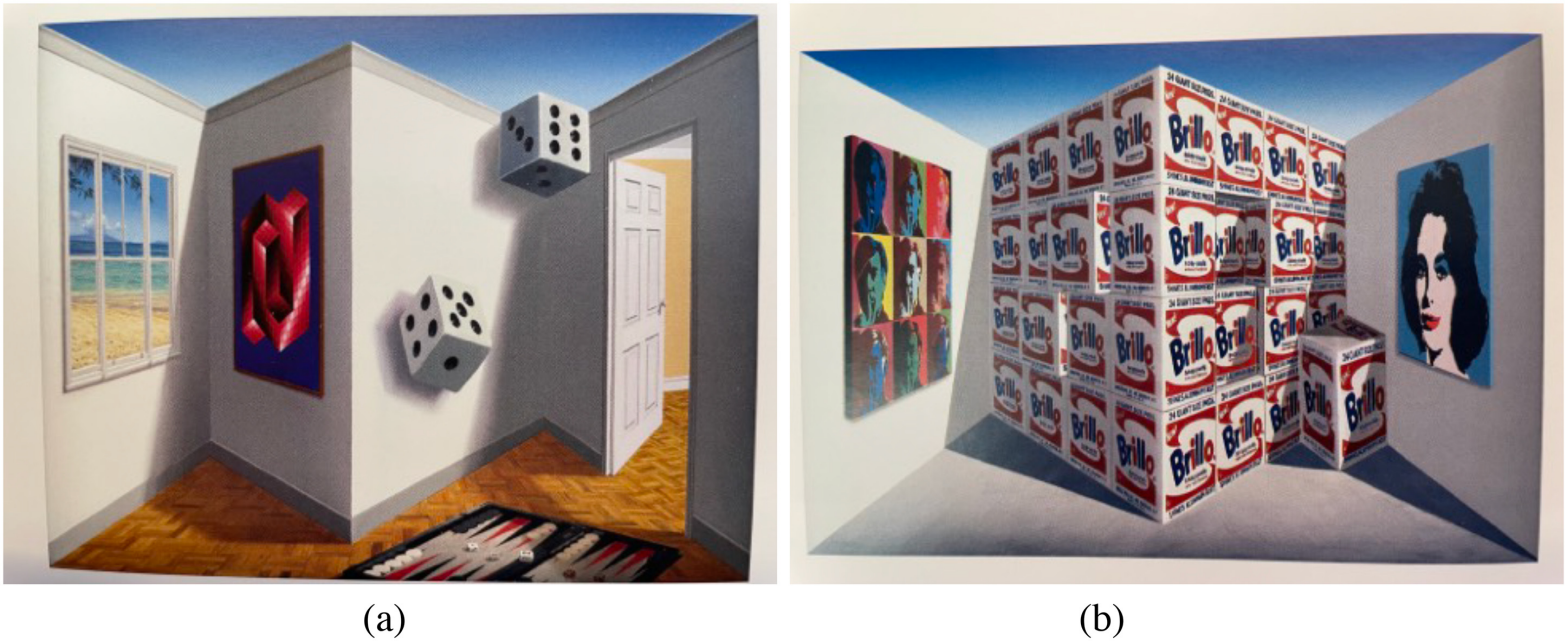
(a) The upper *hollow* die in Patrick Hughes’ “*Dicey*” (2021) is seen as *convex* despite being concave. (The lower die is painted on the wall). (b) The images of the stack of Brillo boxes in his “*Cuboids*” (2021) are painted on the sides of the *valley* between the protruding wedges in the center of the Reverspective. Hence, the large stack of Brillo boxes is seen as protruding *outwards* to the observer because of the strong perspective information.

How does size affect the perception of the Hollow Dice? Consider first the perspective information that is provided by the contours of the Hollow Dice and the gradient of spacing of the dots on the surfaces of the dice (Figure 2). When the angular size of a die is small, the perspective information is very limited. As a result, we can predict that small hollow dice will be seen as convex, not because of the perspective information but rather because of the visual system's “preference” or bias towards seeing 3D forms as convex. And this is what we see when viewing the upper hollow die in “*Dicey*” (Figure 7a).

However, if we increase the angular size of any cuboid structure, the depth increases and the perspective information will eventually dominate—so that the large stack of Brillo boxes in “*Cuboids*” (Figure 7b) is seen as convex despite its physical structure. We think that the critical information that affects our perception of a Reverspective is provided by the perspective present on the images on the *valleys* between the protruding truncated pyramids (or wedges) rather than *peaks* of those protruding truncated pyramids (or wedges). If we think about Reverspectives in this way, there is a strong *similarity* between Reverspectives and hollow faces—the physical structure of the valleys of a Reverspective is *concave* just as the physical structure of a hollow face is *concave*.

For Reverspectives, their concave physical structure (which also determines the motion parallax when the observer moves) is put into conflict with the perspective gradients that are displayed on the valley walls. For hollow faces, their concave physical structure (which also determines the motion parallax when the observer moves) is put into conflict with a bias towards seeing the shading patterns as convex.

## Conflicting Perspectives

**Figure 8.**
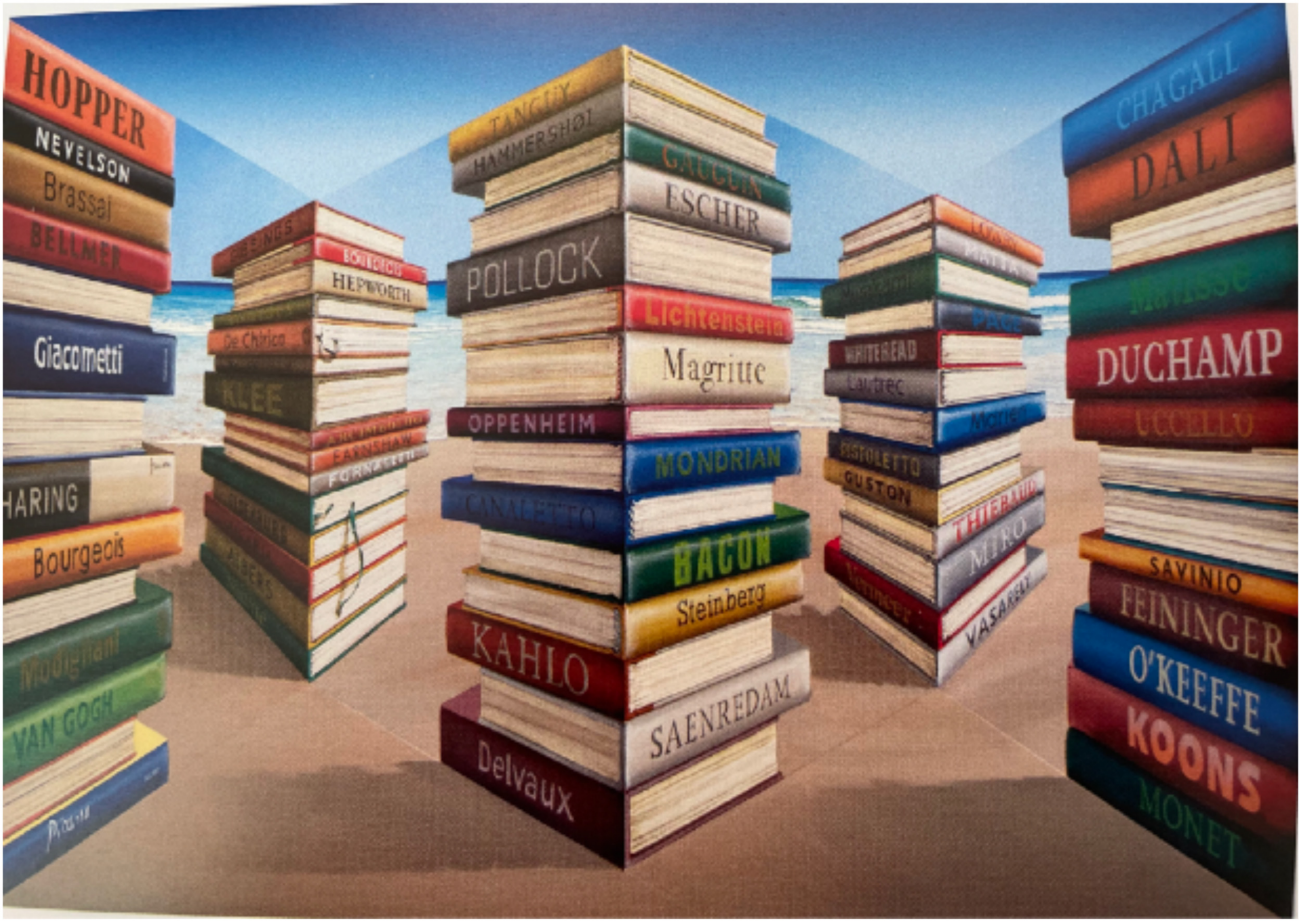
As a result of the perspective in both the larger and the smaller stacks of books in Patrick Hughes’ Reverspective “*Monographs*” (2021), each of the stacks appears to be convex, with the spines of the books receding into the distance. However, when the observer moves from side-to-side (as shown in this video), the larger and the smaller stacks of books appear to rotate in *opposite* directions.

So far, we have attempted to provide an explanation of the perceptual consequences of viewing a Reverspective that is made up of protruding truncated pyramids or wedges like “*Citta Vecchia*” (Figure 1). However, many of Hughes’ more recent Reverspectives are more complex. For example, instead of presenting a single converging perspective gradient from the base to the apex of the wedges as in “*Dicey*” (Figure 7a) and “*Cuboids*” (Figure 7b), “*Monographs*” (2021) splits the perspective information into two parts, both of which provide perspective information: (i) the larger stack of books in the center (that appears to be closer) and (ii) the two smaller stacks of books either side (that appear to be farther away) (Figure 8).

Nothing appears to be unusual to a static observer looking at the Reverspective from a distance—the perspective gradients of both the larger and the smaller stacks provide the information that the spines of the stacks of books are receding into the distance. However, when the observer moves from side-to-side, the larger and smaller stacks of books appear to rotate in *opposite* directions—the central, larger stack of books appears to rotate in the *same* direction as the observer and the two smaller stacks appear to rotate in the *opposite* direction.

How is this possible? Consider an observer who moves from left-to-right. Because the perspective images of the *larger* stack of books (in the center of “*Monographs*”) are on either side of the *valley* between the two protruding wedges, we will see *more* of the left-hand spine of the larger stack (e.g., Pollock) and less of the right-hand spine (e.g., Magritte). As a result, the larger stack of books appears to rotate *counterclockwise,* in the *same* direction as the observer's movement to the right (as shown in Figure 4c). However, during the same observer movement to the right, we will see more of the righthand spines of the *smaller* stacks of books (e.g., Hepworth) and less of the left-hand sides (e.g., De Chirico). As a consequence, the small stack should appear to rotate in a *clockwise* direction*.* And this is what observers report.

**Figure 9.**
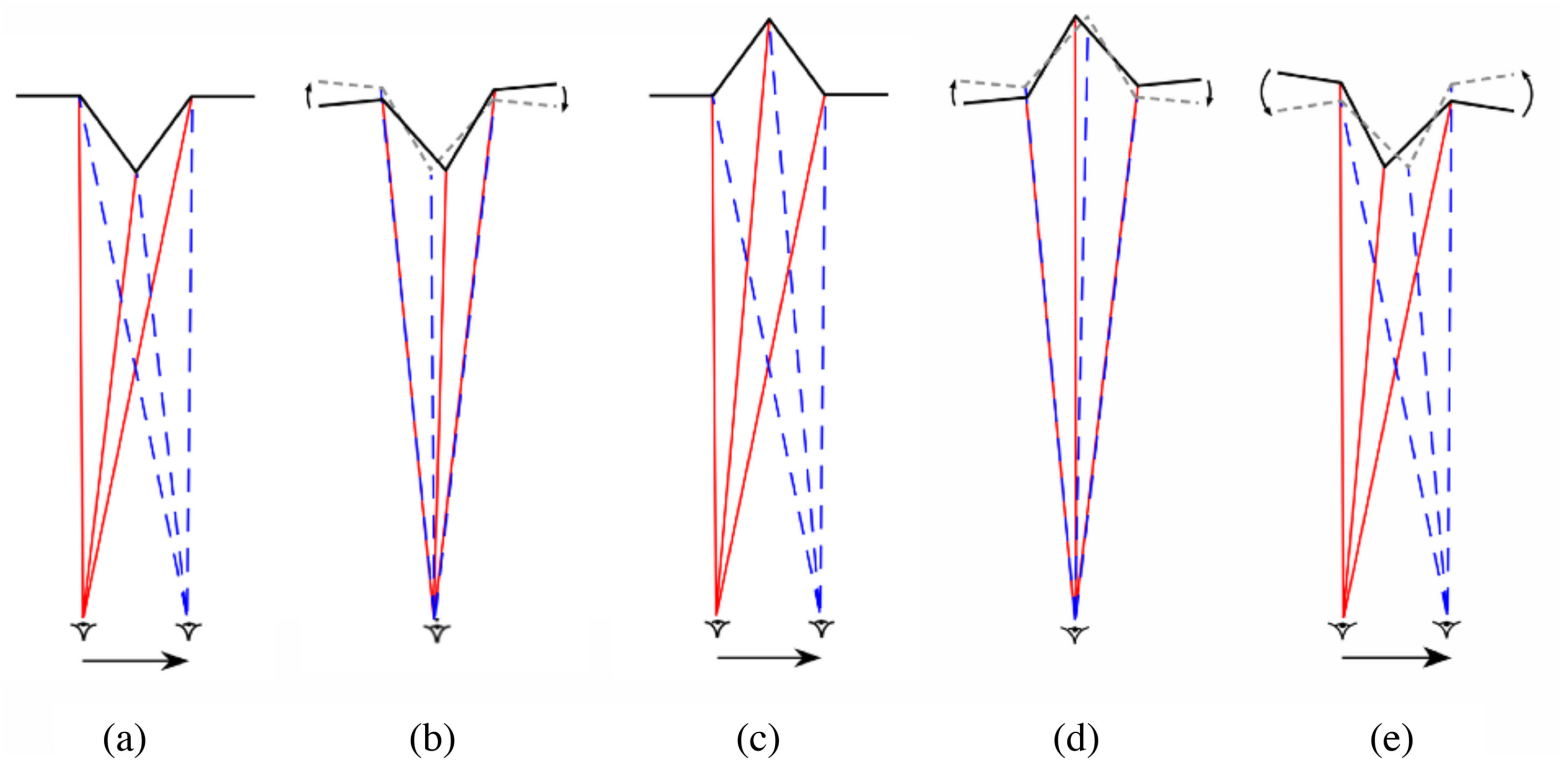
(a) The images of the two *smaller* stacks of books are located on the *peaks* of the protruding wedges such that when the observer moves from left-to-right, the motion parallax is equivalent to a *clockwise* rotation of the small stacks with respect to the line of sight (b). The perspective information on the sides of the peaks is *consistent* with the three-dimensional (3D) structure of the wedges. (c) The images of the *larger* stack of books are located in the concave *valley* between the protruding wedges and this also creates motion parallax that is equivalent to a *clockwise* rotation with respect to the line-of-sight (d). (e) However, the perspective gradients on the sides of the valley signal that the 3D structure of the larger stack of books is *protruding* rather than receding. The only scenario that is consistent with the discrepancy between the parallax created by the valley's 3D structure (c) and the perspective gradients, is to see the larger stack of books rotating in a *counterclockwise* direction (e). [Note that the two superimposed optic arrays (red and blue) depicted in (b) are the *same* as the two optic arrays from different vantage points depicted in (a). Similarly, the two superimposed optic arrays (red and blue) in (d) are the *same* as the two optic arrays from different vantage points depicted in (c)]

How does the perception of the opposite rotations fit with our proposal (outlined earlier) that the perspective gradients on the sides of the truncated pyramids and wedges determine the *sign* of the depth structure and then that sign then determines how the parallax information is “interpreted” or utilized? In “*Monographs*,” the two *smaller* stacks of books are located on the *protruding* peaks of the Reverspective (Figures 8 and 9a) and the perspective gradients on the sides of the peaks also signal their *protruding* 3D shape. Hence there is *agreement* or *complementarity* between the actual 3D structure and the perspective information. When the observer moves from left-to-right, the parallax transformation is equivalent to a *clockwise* rotation of the small stacks with respect to the line-of-sight (Figure 9b) and this is what observers see.

On the other hand, the perspective images of the larger stack of books are located in the *receding* valley of the Reverspective (Figures 8 and 9c) but the perspective gradients on the sides of the valley signal their *protruding* 3D structure. Hence there is a *conflict* between the actual 3D structure and the perspective information. When the observer moves from left-to-right, the motion parallax created by the physical shape of the *receding* valley is equivalent to a *clockwise* rotation with respect to the line-of-sight (Figure 9d). However, the perspective information is dominant and the only scenario that is consistent with the discrepancy between the parallax created by the valley's 3D structure (c) and the perspective gradients, is to see the larger stack of books rotating in a *counterclockwise* direction (e).

As a consequence, the opposite directions of rotation of the larger and smaller stacks of books are entirely consistent with our proposal that the perspective gradients on the sides of the wedges determine the *sign* of the depth structure and that sign then determines how the parallax information is “interpreted” or utilized. In this case, the perspective information is the *same* for the large and small stacks but the motion parallax is in *opposite*^10^ directions and hence the larger and smaller stacks of books appear to rotate in opposite directions.

## Information, Rather Than the Real, Physical Structure is Important

How should we understand the perceptual consequences of viewing the Hollow Dice, the Reverspectives, and the hollow faces? In our view, this is best achieved by considering the available *information,* rather than the actual 3D structures that are presented to the observer. This becomes obvious when we create *virtual* versions of the Hollow Dice, Reverspectives, and hollow faces (Rogers, 2017b). By using 3D display technology or virtual reality we can independently manipulate the different sources of 3D information including the binocular disparities, the perspective gradients, the parallax when the observer moves, and the patterns of shading. As a consequence there is no “real”^11^ depth—there are just different sources of 3D information.^12^ Moreover, it is important to note that there is nothing special about binocular disparities as a source of information, either in theory or in practice. The differences between the two binocular images are simply a consequence of the two different *perspective* views of a 3D scene, just as the motion parallax transformations created when an observer moves are a consequence of the *changing* perspective image over time.

However, by manipulating the *features* of displayed images—whether those features are random texture patterns, oriented line segments, smoothly changing shading patterns, familiar objects, or natural images—we can determine which information is used by the visual system. For example, if a hollow face is covered with a pattern of high-contrast random dots, those random dots provide disparity information (for a binocular observer) that specifies the “real” concave shape of the face. As a consequence, the face can be seen as hollow (Georgeson, 1979). Similarly, if the Hollow Dice were covered with a pattern of random dots (thereby creating binocular disparities to specify their “real” concave shape), we might expect that the Dice would also be seen as concave. However, when we tried this, there was little or no difference in the “flipping distance^13^” when the Hollow Dice were viewed *binocularly*. This is perhaps not surprising since our Dice were smaller than Georgeson's hollow head, and created a smaller disparity difference (∼ 9 arc min) between the front and back of each Die at the 130–160 cm viewing distance. A further benefit of considering the *information* that is available in any particular situation (rather than the physical structure) is that once we have identified (precisely) the information that is used by the visual system, we have effectively described the characteristics and functioning of the underlying mechanisms (Gibson, 1979).

## Conclusions

We can all enjoy the pleasure and delight of viewing Reverspectives and Hollow Dice as pieces of unusual and original art but at the same time, their unique structures have provided us with a valuable experimental tool for understanding the workings of the visual system. We think that by describing the input to the perceptual system in terms of the different sources of *information,* rather than their actual 3D structure,^14^ we can better understand how and why we see Reverspectives, Hollow Dice, and hollow faces in the way that we do—their reversed depth; their perceived rotation when the observer moves; and their different appearances under monocular versus binocular viewing. You can call all three perceptual effects *illusions* if you like but what they reveal is how the perceptual system functions when faced with different and sometimes conflicting sources of 3D information.

## Notes

1. We have used quotation marks around the word “interpretation” because it suggests that there is some higher-level or cognitive process of interpretation. In this article, we use the word to describe any perceptual situation where there are alternative perceptual outcomes.

2. That is, they are *less* likely to the be seen as convex.

3. Richard Gregory (who liked wordplays and puns) coined the phrase “flipping” distance (Gregory, 2005).

4. Papathomas and Bono (2004) found that the “flipping” distance was closer to a Reverspective than to a hollow face under both monocular and binocular viewing conditions.

5. Readers may have noticed that the surrounding landscape appears to rotate around the point of fixation when viewed from a moving car or train.

6. C*lockwise* with respect to the observer's *line-of-sight* is equivalent to *stationary* with respect to the *world.*

7. The perspective information is minimal because the difference in depth between the front and back of the dice is small (5–10 cm).

8. Depending on the viewing distance.

9. It is an empirical question as to whether we perceive this particular difference in depth.

10. The differences between the changing optic arrays can be seen in Figure 9b and d.

11. We have used quotation marks around the word “real” because there is nothing special about the *real* structure of Reverspectives, Hollow Dice and hollow faces. (Demo at the 2013 ECVP meeting in Bremen).

12. For example, we can simulate a Reverspective with disparities that specify a *flat* surface (thereby mimicking a flat painting) but with motion parallax equivalent to the truncated pyramids and wedges.

13. (Between 130 and 160 cm).

14. The same point has been made about the Ames Room (Rogers, 2022). The actual trapezoidal shape of the Ames Room is irrelevant. What we see is consistent with the perspective information.
